# Evaluation of radiologists’ accuracy and interrater reliability when interpreting CT scans after ventral hernia repair

**DOI:** 10.1007/s10029-025-03504-7

**Published:** 2025-10-31

**Authors:** Fahim Kanani, Rina Neeman, Narmin Zoabi, Elad Karin, Bar Cohen, Eran Nizri, Adam Abu Abeid, Yonatan Lessing, Guy Lahat, Michael J. Rosen, Nir Messer

**Affiliations:** 1 Department of Surgery, Tel Aviv Medical Center, Tel Aviv, Israel; 2 Department of Radiology, Tel Aviv Medical Center, Tel Aviv, Israel; 3 Department of Surgery, Wolfson Medical Center, Tel Aviv, Israel; 4 Department of Gastroenterology, Sheba Medical Center, Ramat Gan, Israel; 5 Division of GI Surgery, Northwestern University, Chicago, IL US

**Keywords:** Ventral hernia repair, Computed tomography (CT), Interpretation, Accuracy, Interrater reliability, Reoperation, Mesh plane, Mesh type

## Abstract

**Background:**

Accurate interpretation of computed tomography (CT) prior to ventral hernia repair (VHR) is critical for operative planning, particularly in recurrent cases where prior mesh type and placement influence surgical approach. While radiologists possess specialized expertise in imaging interpretation, their performance in identifying post-VHR mesh characteristics remains unstudied. This study assesses the diagnostic performance of radiologists in identifying mesh plane and type on CT following VHR.

**Methods:**

Forty body radiologists from 16 centers evaluated 18 de-identified CT scans, including 12 post-VHR cases and 6 controls. After receiving standardized training on mesh characteristics and surgical planes, radiologists were tasked with identifying both the VHR mesh plane and the mesh type. The study assessed correct identification rates, interrater reliability, and repeatability.

**Results:**

Radiologists demonstrated overall accuracy rates of 39.2% for mesh plane identification and 43.5% for mesh type identification. Accuracy was highest for intraperitoneal approaches (53.8%) and heavy-weight mesh (52.5%), with significantly lower accuracy (16.9–22.5%) for other surgical planes and mesh types (30.6%). Interrater reliability was poor (Gwet’s AC1 = 0.127), with significant between-radiologist variability. Self-reported confidence levels correlated positively with accuracy rates, with high-confidence radiologists showing significantly better performance (OR 4.55, *p* < 0.001). Years of clinical experience did not predict diagnostic accuracy.

**Conclusions:**

Radiologists demonstrated limited ability to accurately interpret post-VHR CT scans, even after targeted instruction. These diagnostic challenges mirror findings among abdominal wall reconstruction surgeons, suggesting inherent limitations in CT-based assessment rather than discipline-specific deficits. Multidisciplinary collaboration, standardized operative documentation, and dedicated abdominal wall imaging protocol and training are essential to improve diagnostic accuracy and optimize surgical planning.

**Supplementary Information:**

The online version contains supplementary material available at 10.1007/s10029-025-03504-7.

## Introduction

 Addressing recurrent ventral hernias presents significant surgical challenges, primarily due to the necessity of revisiting planes disrupted during prior operations, and the presence of preexisting mesh which often impedes surgical access [1]. These factors inherently increase the complexity of the procedure, leading to prolonged operative times, heightened risks of morbidity, and elevated recurrence rates [2, 3]. Proper planning for recurrent ventral hernia repair (VHR) requires a detailed understanding of the patient’s surgical history, particularly the mesh plane and the type of mesh previously used. This knowledge enables surgeons to plan the most suitable operative approach and technique, determine the appropriate mesh plane and type, anticipate surgical complexity, and accurately estimate operative duration. For example, a prior repair with a medium-weight mesh and a central defect might indicate a mesh fracture, prompting the use of heavier material for the next repair [4]. Similarly, if a biologic mesh was used, it may have degraded, eliminating the need for removal. In some cases, large defects may necessitate advanced techniques to improve outcomes in abdominal wall reconstruction. Moreover, understanding the history of previous repairs aids in guiding patient and family counseling, helping to set realistic expectations.

Despite its critical role, accurate surgical planning is often hindered by incomplete or unavailable historical data regarding prior interventions, mesh characteristics, and dissection planes [5, 6]. Retrieving surgical records is often time-consuming and frequently unproductive, particularly in resource-limited settings or urgent cases [7]. To address these challenges, CT imaging has emerged as an indispensable tool, providing critical insights into prior procedures and anatomical configurations [8]. While CT imaging bridges documentation gaps, its effectiveness is constrained by variability in interpretative accuracy [4, 9].

The evaluation of prior abdominal wall surgeries and their associated implants is currently performed predominantly by abdominal wall reconstruction surgeons [4, 10].However, recent studies highlight significant limitations in surgeons’ ability to accurately interpret post-VHR CT scans, with prior repair techniques and mesh types correctly identified in fewer than 50% of cases [1, 5–8, 10, 11]. This underscores the need for enhanced imaging interpretation to optimize surgical planning. Given their specialized expertise, radiologists may improve the accuracy of complex CT scan assessments. However, in our experience, when abdominal CT scans are obtained to evaluate abdominal wall anatomy prior to recurrent VHR, radiologists often allocate minimal attention to the abdominal wall and hernia defect. While not formally studied, this observation has been anecdotally reported by other high-volume AWR centers [11].

To evaluate radiologists’ precision, we assessed their ability to identify the mesh plane and type implanted during prior VHR, following targeted guidance on the anatomical planes and characteristic appearances of various mesh types.

## Methods

Following approval from the Institutional Review Board (IRB), 40 expert body radiologists from 16 centers specializing in abdominal wall reconstruction (AWR) in North America (United States and Canada), Western Europe (Germany, United Kingdom, France, and Spain), Australia and Asia (Israel) were enrolled to evaluate 18 CT scans: 12 from patients who underwent VHR with permanent synthetic mesh and 6 controls (3 with laparotomy without hernia repair and 3 with no prior abdominal surgery).

**Fig. 1:**
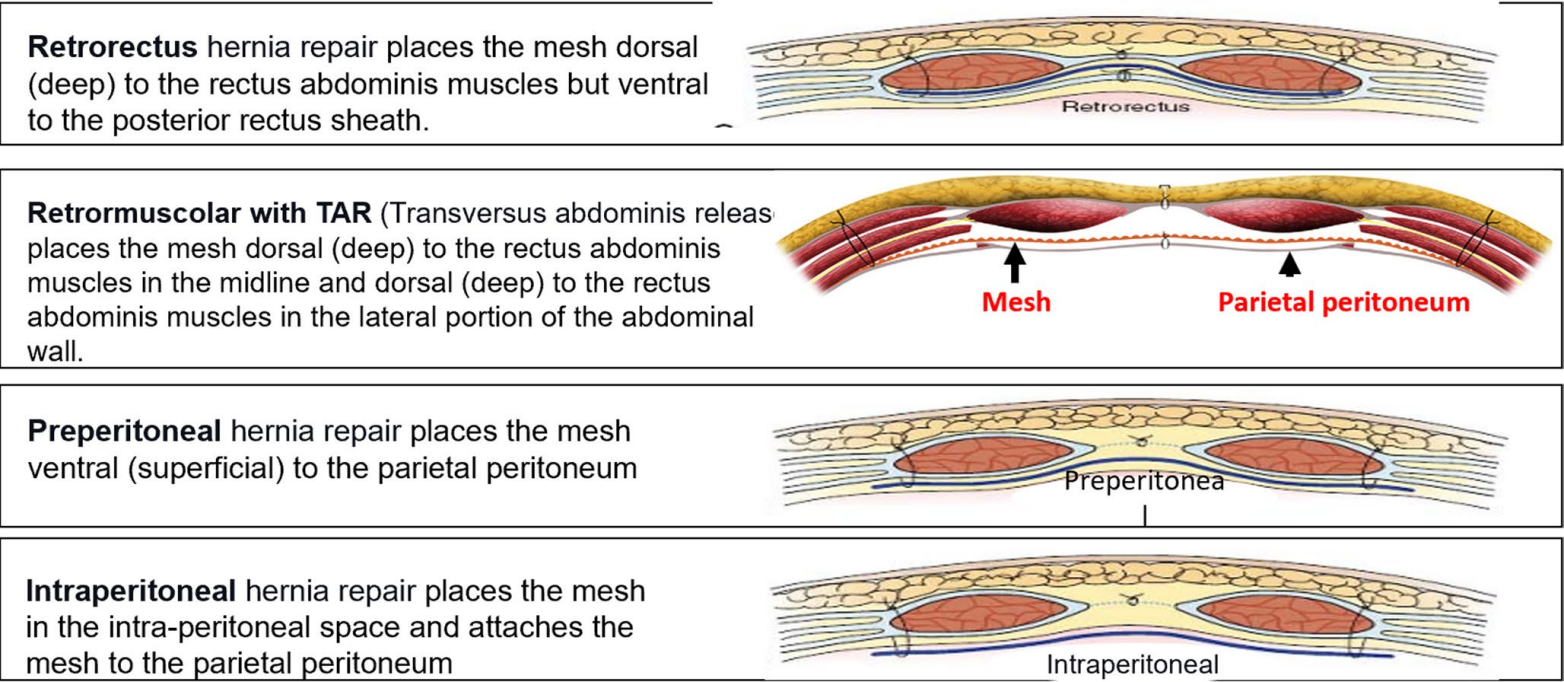
Mesh plane terminology follows the EHS International Classification of Abdominal Wall Planes (ICAP)

**Fig. 2:**
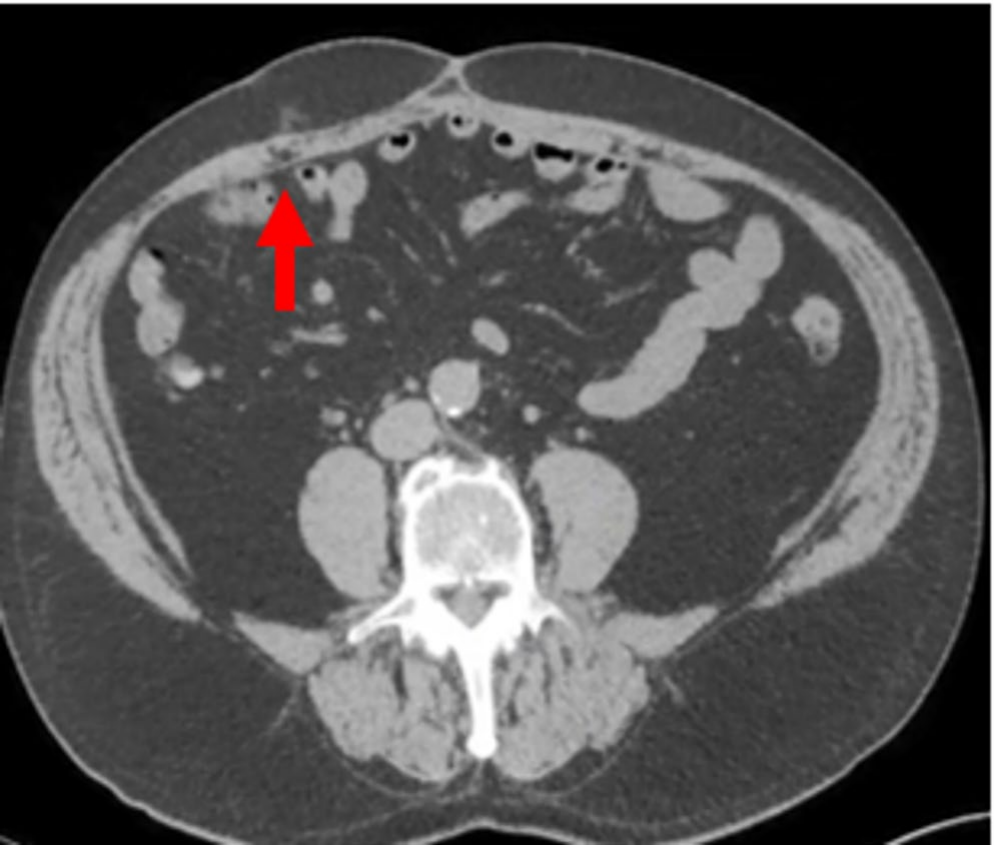
Heavy-Weight Mesh

**Fig. 3:**
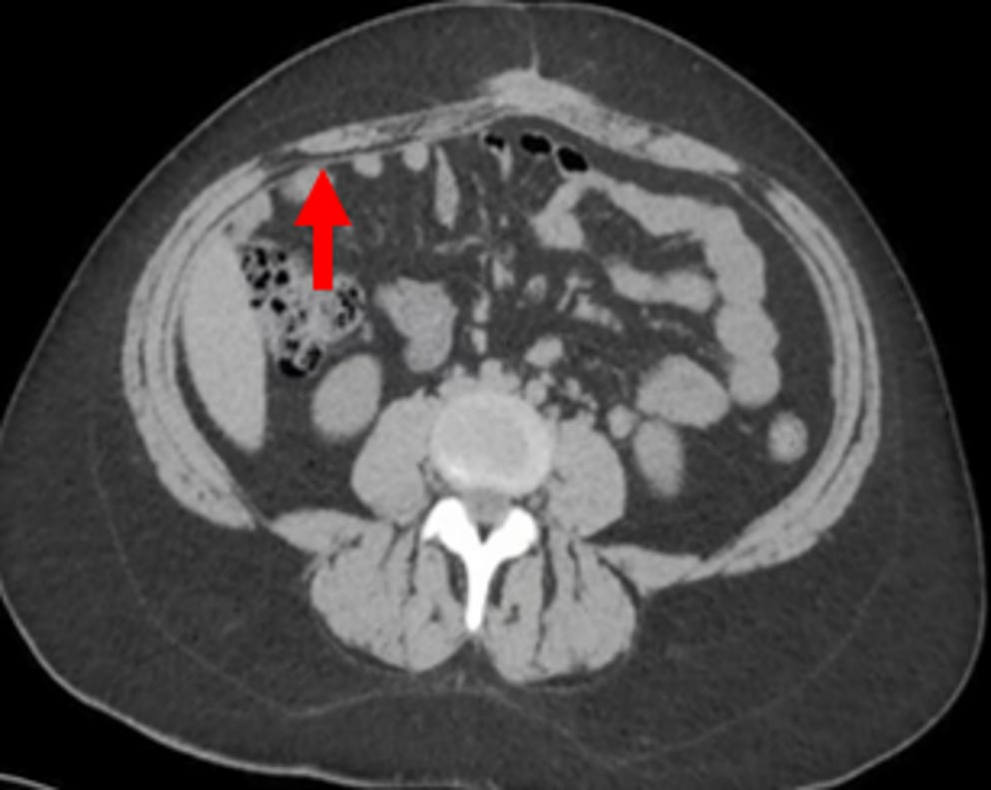
Medium Weight Mesh

**Fig. 4:**
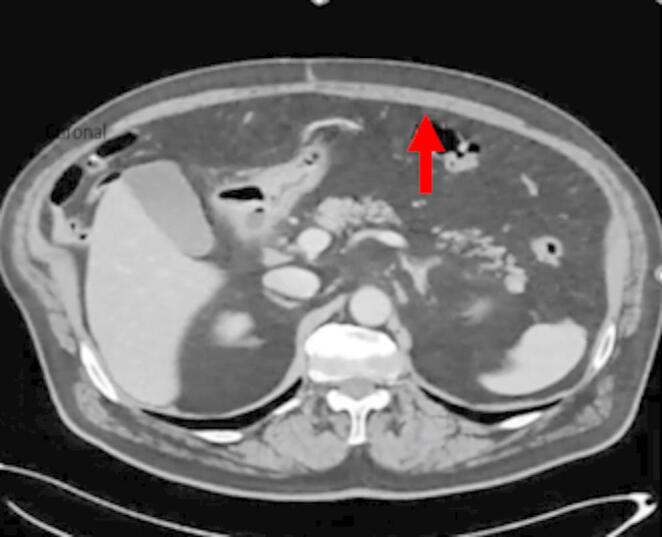
Light Weight Mesh

All participating radiologists were subspecialty-trained in body CT with extensive experience in postoperative CT interpretation, including a minimum of five VHR-related studies weekly. Each radiologist independently assessed 18 CT scans performed two years post-VHR. The 2- year post-operative timepoint was selected based on several considerations: (1) complete mesh incorporation and tissue remodeling typically occurs by 12–24 months, providing stable imaging characteristics; (2) this timeframe represents when many patients present with recurrent hernias requiring reoperation; and (3) it minimizes acute post-operative changes that could confound mesh visualization while maintaining clinical relevance for surgical planning. Radiologists completed a structured questionnaire to specify mesh location and type. The VHR planes were classified according to the European Hernia Society’s International Classification of Abdominal Wall Planes (ICAP) (Parker SG, et al. International classification of abdominal wall planes (ICAP) to describe mesh insertion for ventral hernia repair. Br J Surg. 2020;107(3):209–217). The planes assessed included: (1) retrorectus plane - mesh positioned posterior to the rectus muscle and anterior to the posterior rectus sheath; (2) retromuscular plane - accessed only after transversus abdominis release (TAR), with mesh extending laterally beyond the semilunar line; (3) preperitoneal plane - mesh positioned between the peritoneum and transversalis fascia; and (4) intraperitoneal plane - mesh placed within the peritoneal cavity (Fig. 1). All meshes used in this study were permanent synthetic polypropylene and categorized by density. Heavy-weight synthetic mesh (HWSM) was defined as PROLENE^®^ Mesh, manufactured by Ethicon with a density of 95 g/m² (Fig. 2). Medium-weight synthetic mesh (MWSM) referred to Bard™ Soft Mesh, with a density of 43 g/m², and light-weight synthetic mesh (LWSM) corresponded to Ultrapro™ advanced, with a weight of 26 g/m² (Figs. 3 and 4) Detailed characteristics for all 18 cases, including mesh specifications and CT parameters, are provided in Supplementary Table 1. Additionally, the study incorporated CT scans from two control groups: a negative control group with patients who had never undergone abdominal surgery, and a positive control group with patients who had undergone laparotomy without VHR. Control CT scans were obtained at variable timepoints: positive control scans (laparotomy without VHR) were acquired 3–6 months postoperatively to capture healed surgical changes without mesh, while negative control scans (no prior abdominal surgery) had no timing restrictions as they served to demonstrate normal abdominal wall anatomy. The 3–6 month timepoint for positive controls was selected to ensure complete laparotomy healing while avoiding long-term tissue remodeling that might obscure surgical plane distinction. While this creates temporal mismatch with 24-month VHR scans, the primary diagnostic task focuses on mesh presence/absence rather than temporal evolution of surgical changes. This timepoint captures healed surgical changes without mesh-related artifacts, providing appropriate control for radiologist assessment. All radiologists were informed of the inclusion of these control scans in the study.

The electronic medical record (EMR) system was utilized to identify patients who had undergone VHR. FK and NM conducted a manual chart review to examine each patient’s operative reports, verify the VHR technique, plane, and type of mesh used, and select a CT scan that did not include cases with complications such as surgical site occurrences (SSO) or surgical site infections (SSI); Moreover, the CT scans didn’t include multiple meshes in the different planes. Instead, the study exclusively used straightforward surgeries without recurrences within two years of an uneventful postoperative course. CT scans for the control arms were selected through a manual chart review of EMR. For the negative control group, all operative reports and history and physical (H&P) notes were reviewed to confirm the absence of prior abdominal surgery. For the positive control group, similar reviews were conducted to verify the absence of VHR. Where available, operative reports from external institutions were also examined to ensure eligibility. All personal health information (PHI) was anonymized to ensure confidentiality. All radiologists were informed on the inclusion and exclusion criteria of the CT scans in the study.

**Scheme 1:**
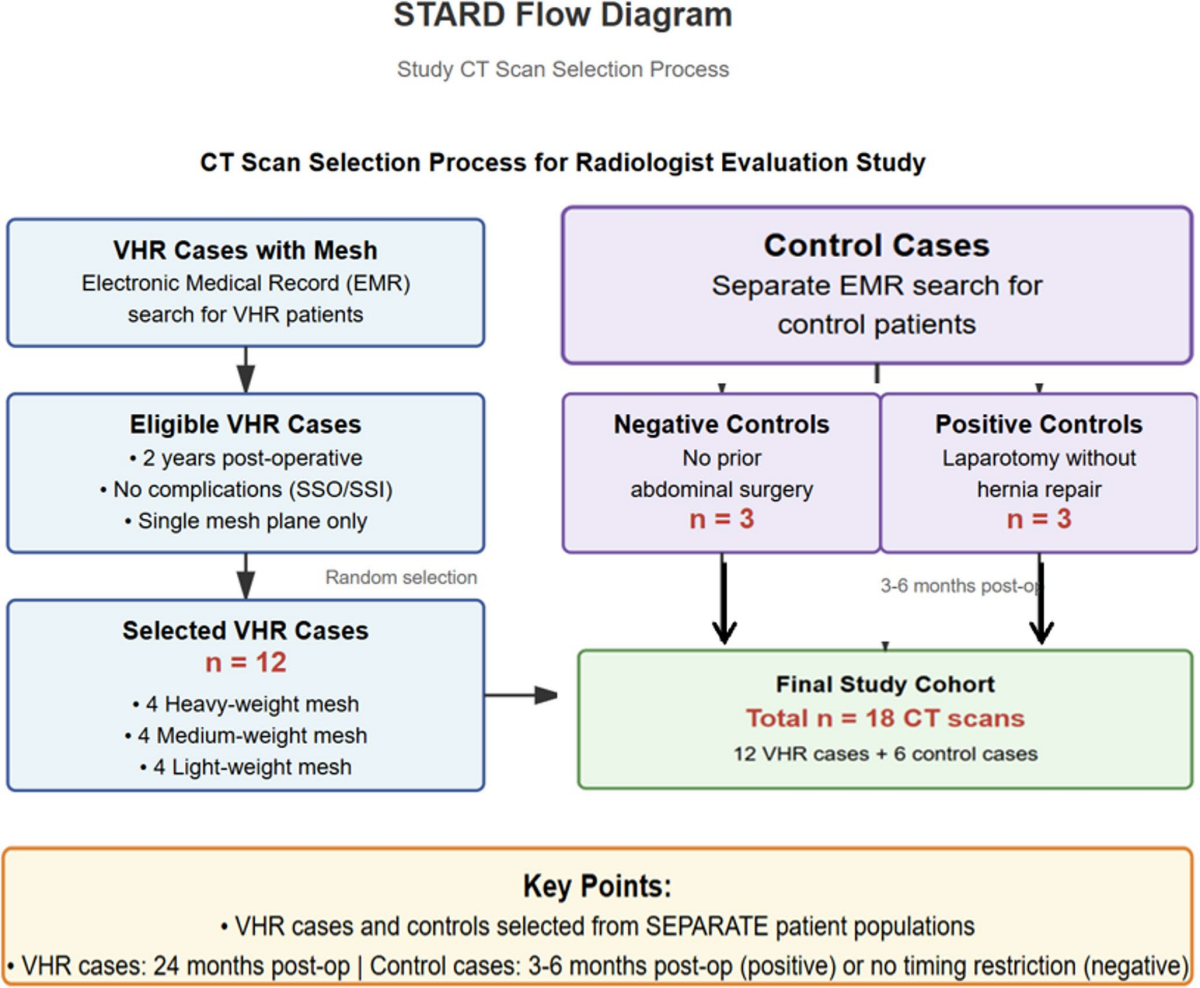
STARD flow diagram for CT scan selection. Two independent pathways show selection of 12 VHR cases with mesh and 6 control cases (3 negative, 3 positive), yielding 18 total CT scans for radiologist evaluation. EMR = Electronic Medical Record; VHR = Ventral Hernia Repair

Upon obtaining participants’ consent, an informational institutional email was sent explaining the questionnaire process. The email included a Microsoft PowerPoint file containing 18 CT scan videos with axial, coronal, and sagittal views. These comprised four scans for each mesh type (HWSM, MWSM, and LWSM), along with six control scans (three negative [no surgery] and three positives [surgery without hernia repair]). Participants could freely scroll through the CT videos in the presentation to assess the plane and mesh type without time constraints. All included CT scans met standardized technical parameters: 5 mm slice thickness, standard soft tissue reconstruction kernel, and availability of multiplanar reformats. Computer randomization was used to select the 12 study cases from the eligible cohort, maintaining equal distribution across mesh. The case selection process followed STARD guidelines and involved two separate pathways (Scheme 1). For VHR cases, we searched our EMR system to identify patients who had undergone ventral hernia repair with mesh placement, ultimately selecting 12 cases. Control cases were selected through a separate EMR search of patients without ventral hernia repair, yielding 6 controls (3 negative, 3 positive). The final study cohort comprised 18 CT scans total.

To ensure a standardized baseline understanding of surgical techniques and mesh characteristics, all participating radiologists received a comprehensive 30-minute instructional session before evaluating the CT scans. This standardized training protocol was delivered individually via video conference by the lead investigator (FK) with three specific learning objectives: (1) accurately identify the four anatomical planes used for mesh placement, (2) recognize radiographic density characteristics distinguishing heavy, medium, and light-weight meshes, and (3) differentiate normal post-operative changes from mesh-related findings. The training presentation included detailed anatomical illustrations with arrows indicating precise mesh placement in each surgical plane (Fig. 1), representative CT examples of each mesh type validated against operative reports as reference standards, and clear definitions of mesh weight categories with their corresponding densities (heavy-weight: 95 g/m², medium-weight: 43 g/m², light-weight: 26 g/m²). Following the training session, participants proceeded directly to case evaluation. While we did not implement formal pre/post-training competency testing, participants were encouraged to ask clarifying questions to confirm understanding and remained unaware whether they were evaluating pilot or study cases, maintaining blinding throughout. The complete standardized training presentation used for all participants is available via link in the supplementary materials, allowing verification of instructional content and terminology consistency with ICAP classification.

Data was collected through a web-based questionnaire using the Google Forms platform (USA), designed for self-completion by individual participants. The questionnaire was divided into two sections: A section (questions 1–3 and 40–44) gathering general professional information on the participants’ experience in abdominal wall CT imaging using a 10-point Likert scale (1 = lowest, 10 = highest) (Table 1), And a section (questions 4–39) focused on the evaluation of the 18 CT scans. A pilot test was conducted with 10 radiology residents to refine the presentation of the CT scans and optimize the questionnaire design. Notably, the CT scans used in the pilot test were distinct from those used in the main study. After revisions based on pilot feedback, the final questionnaire was distributed to study participants.

**Table 1:** Demographic and experience characteristics of radiologist participants (*N* = 40)

Question/Characteristic	Mean	Median	IQR	Distribution (Score: *n* (%))
Post-training experience (years)	4.61	3	5	1 Year: 10 (25%) 1–5 Years: 11 (27.5%) 5–7 Years: 11 (27.5%) 7–10 Years: 3 (7.5%) 10 + Years: 5 (12.5%)
Ventral hernia CT scans per week	13.75	15	10	Less than 10: 15 (37.5%) 10 to 20: 15 (37.5%) More than 20: 10 (25%)
How familiar were you with mesh planes?	3.7	4/10	2	Low (< 4): 13 (32.5%), Moderate (4–8): 27 (67.5%), High (> 8): 0 (0.0%)
How familiar were you with mesh types?	3.75	4/10	2	Low (< 4): 12 (30.0%), Moderate (4–8): 28 (70.0%), High (> 8): 0 (0.0%)
How confident were you in your responses?	4.7	4/10	2	Low (≤ 2): 3 (7.5%), Moderate (4–6): 34 (85.0%), High (≥ 8): 3 (7.5%)
Need for hernia-specific training?	9.15	10/10	2	Moderate (6): 3 (7.5%), High (8): 11 (27.5%), Very High (10): 26 (65.0%)

Table 1: Provides a complete overview of your radiologist participants, including their experience levels, comfort with hernia interpretation, and need for specialized training

The study’s primary outcome was the accuracy rate, reflecting the radiologists’ ability to interpret CT scans correctly. Answering two questions per scan: one regarding the identification of the mesh plane, and the other concerning the type (density) of mesh used. For mesh plane identification, the accuracy rate was calculated based on 480 responses (40 radiologists × 12 VHR scans) and 240 control responses (40 radiologists × 6 control scans). Similarly, for mesh type identification, the accuracy rate was derived from 480 responses for VHR scans and 240 responses for controls.

In addition to accuracy, interrater reliability was assessed to evaluate the level of agreement among radiologists, regardless of response accuracy. Repeatability (R) was also analyzed to determine the consistency of individual radiologists in identifying the same VHR plane and mesh type across repeated evaluations.

Accuracy was calculated as the percentage of correct CT scan identifications for each VHR plane and mesh type. To analyze diagnostic accuracy across categories, we employed a generalized linear mixed-effects model (GLMM) with a log link function. This statistical approach combines fixed effects (variables consistent across all data) and random effects (variables that account for variations across data), making it suitable for handling correlated or non-independent data, such as repeated measures or hierarchical structures. The model was chosen because multiple CT scans were evaluated by the same radiologists, necessitating adjustments for repeated measurements. Radiologist-specific random effects were included to adjust for individual differences in diagnostic performance, such as variations in accuracy, consistency, or interpretative approaches. This ensured that the variability between radiologists did not bias the overall results while capturing their unique assessment patterns. Results are expressed as Incident Rate Ratios (IRRs), where values above 1.0 indicate easier identification and values below 1.0 signify more difficult identification. All comparisons were made relative to a negative control accuracy rate as the reference. Sensitivity analyses examined case-level difficulty patterns and confirmed that within-plane consistency was high, supporting our decision to model reader-level random effects while capturing case difficulty through fixed effects for mesh planes. The dominant source of variability was between readers rather than between cases within the same mesh plane category.

Agreement between radiologists was evaluated using two measures: Gwet’s AC1 for inter-rater reliability and Intraclass Correlation Coefficient (ICC) for repeatability. Gwet’s AC1 coefficient was selected for inter-rater reliability assessment as both outcome variables (mesh plane and mesh type) represent nominal categories without inherent ordering. AC1 provides a chance-corrected agreement measure that is more robust to prevalence and marginal probability imbalances than traditional kappa statistics, making it particularly suitable for our multi-category classification task. Gwet’s AC1 coefficients measure agreement between radiologists regardless of correctness, and were interpreted as: poor (< 0.2), fair (0.2–0.4), moderate (0.4–0.6), good (0.6–0.8), and very good (0.8–1.0.8.0). The ICC, which measures repeatability, ranges from 0 to 1, where 1 indicates perfect repeatability and lower values indicate decreased consistency (Table 5 Part B). Specifically, we calculated ICC(3,1) - a two-way mixed-effects model with consistency definition for single raters, appropriate when specific raters (not randomly sampled) evaluate random cases and individual rater reliability is the clinical reality. We performed two distinct correlation analyses using Pearson’s chi-square test. First, we examined whether there was a correlation between radiologists’ self-reported confidence level (rated on a scale of 1–10) and their diagnostic accuracy rates. In our second analysis, we investigated the potential relationship between radiologists’ performance on negative control cases and their overall diagnostic accuracy. For this second analysis, radiologists were stratified into four categories according to their success rate in interpreting negative control CT scans, ranging from no correct interpretations to all three negative control cases interpreted correctly. Statistical methods were designed and implemented by team members with formal biostatistics training, with oversight from academic faculty as needed for complex analyses. All statistical analyses were performed using SPSS (version 29) and R (version 4.2.3).

## Results

Following the case selection process outlined in Scheme 1, a cohort of 40 radiologists with specialized expertise in body CT participated in this study, with a median post-training experience of 3 years (IQR: 5 years; mean: 4.61 years). The participants reported interpreting a median of 15 ventral hernia CT scans per week (IQR: 10). Regarding familiarity with mesh plane and mesh type before the illustrative pre-study explanation, median scores were 4/10 (IQR: 2) and 4/10 (IQR: 2), respectively. When assessing their confidence in evaluating ventral hernia CT scans with a focus on abdominal wall assessment, radiologists reported a median confidence score of 4/10 (IQR: 2). Participants’ baseline experience is detailed in Table 1.

Table 2 outlines the accuracy of mesh plane identification, defined as the rate of correct identification of the mesh plane. The overall accuracy for correctly identifying the mesh plane was 39.2%. Accuracy was highest in cases involving the intraperitoneal mesh plane, with a correct identification rate of 53.8%. This was followed by preperitoneal plane (22.5%), retrorectus plane with extension beyond the semilunar line (18.8%), and retrorectus plane without extension (16.9%). Negative controls were correctly identified in 55.8% of cases, while positive controls were accurately identified in 30.0% of cases.

**Table 2:** Mesh plane identification accuracy

Mesh Plane	Total Cases	Successful Identifications	Success Rate
Intraperitoneal	160	86	53.8%
Retromuscular with TAR	80	30	18.8%
Retrorectus	80	27	16.9%
Preperitoneal	160	36	22.5%
No abdominal surgery (Negative control)	120	67	55.8%
Laparotomy without hernia repair (Positive control)	120	36	30.0%
Overall accuracy for mesh planes	**720**	**282**	**39.2%**

Table 2. Shows the success rates for identifying different mesh planes, highlighting the disparity between intraperitoneal placement (53.8%) and other approaches (16.9–22.5.9.5%)

Table 3 presents the accuracy of mesh type identification. The overall accuracy for correctly identifying the mesh type was 43.5%. Among the various mesh types, accuracy was highest in cases involving heavy-weight mesh, with a correct identification rate of 52.5%, followed by medium-weight and light-weight mesh with identical accuracy rates of 30.6%. Across control cases, 54.6% of radiologists correctly identified the absence of mesh.

**Table 3:** Mesh type identification accuracy

Mesh Type	Total Cases	Successful Identifications	Success Rate
Heavy-weight mesh	160	84	52.5%
Medium-weight mesh	160	49	30.6%
Light-weight mesh	160	49	30.6%
No mesh*	240	131	54.6%
Overall accuracy for mesh types	**720**	**313**	**43.5%**

Table 3. Presents the accuracy rates for different mesh types, showing heavy-weight mesh (52.5%) was more readily identified than medium/light-weight options (30.6%)

*The "No mesh" success rate represents the average of negative and positive controls

Table 4 displays the distribution of responses for mesh plane identification, revealing specific patterns of misidentification. Notably, preperitoneal mesh placement was misclassified as intraperitoneal in 29% of cases, while retrorectus with TAR was most commonly misidentified as preperitoneal (37.5%). This detailed analysis highlights where diagnostic confusion most frequently occurs, with implications for surgical planning.

**Table 4:** Distribution of responses for mesh plane identification (*N* = 720)

True Plane/Identified as:	Intraperitoneal	Retrorectus	Retromuscular with TAR	Preperitoneal	Positive control	Negative control	Total
Intraperitoneal	**86 (53.8%)**	17 (10.6%)	28 (17.5%)	24 (15.0%)	2 (1.3%)	3 (1.9%)	160 (100%)
Retrorectus	16 (20.0%)	**17 (21.3%)**	12 (15.0%)	27 (33.8%)	3 (3.8%)	5 (6.3%)	80 (100%)
Retromuscular with TAR	14 (17.5%)	10 (12.5%)	**18 (22.5%)**	28 (35.0%)	6 (7.5%)	4 (5.0%)	80 (100%)
Preperitoneal	46 (28.7%)	20 (12.5%)	25 (15.6%)	**36 (22.5%)**	20 (12.5%)	13 (8.1%)	160 (100%)
Positive control	12 (10.0%)	14 (11.7%)	16 (13.3%)	20 (16.7%)	**36 (30.0%)**	22 (18.3%)	120 (100%)
Negative control	8 (6.7%)	9 (7.5%)	7 (5.8%)	13 (10.8%)	16 (13.3%)	**67 (55.8%)**	120 (100%)
Total	182(25.3%)	87(12.1%)	106(14.7%)	148(20.6%)	83(11.5%)	114(11.5%)	**720(100%)**

Table 4. Reveals detailed patterns of misidentification, demonstrating where radiologists most commonly made errors (e.g., misclassifying preperitoneal as intraperitoneal)

*Numbers in bold represent correct identifications, p<0.001

**Table 5:** Accuracy assessment using incident rate ratio and interrater reliability part A: mesh plane identification accuracy (Reference: negative Control)

Part A: Mesh Plane Identification Accuracy (Reference: Negative Control)						
Parameter	Incident rate ratio	SE	Confidence interval	z	*p*-value	
Intraperitoneal	0.964	0.084	(0.81, 1.15)	−0.42	0.678	
Pre-peritoneal	0.403	0.062	(0.29, 0.55)	−5.85	< 0.001	
Retromuscular with TAR	0.337	0.058	(0.24, 0.48)	−7.09	< 0.001	
Retrorectus	0.303	0.055	(0.21, 0.44)	−7.93	< 0.001	
Part B: Interrater Reliability Analysis						
Analysis Type	Measure	Value	Std. Error	Z/Coeff.SE	*P*-value	95% CI
Random Effect	Variance (expert_n)	0.480	0.182	2.645	0.008	0.229–1.008
Agreement	Gwet’s AC1	0.127	0.026	-	< 0.001	0.073–0.180
ICC - Mesh Type	0.360	-	-	-	-	
ICC - Mesh Plane	0.270	-	-	-	-	
IRR - Mesh Type	54.970	-	-	-	-	
IRR - Mesh Plane	37.350	-	-	-	-	

Table 5. A Combines statistical analysis of accuracy with reliability measures, showing both the relative difficulty of identifying different planes and the consistency of interpretations

*IRR *Incident rate ratios, *ICC* Intraclass Correlation Coefficient; IRR values represent incident rate ratios (not percentages). These indicate the relative rate of correct identification compared to the reference category

The accuracy assessment of mesh plane and type, evaluated using incident rate ratio (IRR), is detailed in Table 5 (Part A). Identification of the intraperitoneal mesh position was comparable to negative controls, with an IRR of 0.964 (95% CI: 0.81–1.15, *p* = 0.678). However, accuracy decreased significantly for preperitoneal placement (IRR: 0.403, 95% CI: 0.29–0.55, *p* < 0.001), retromuscular placement with TAR (IRR: 0.337, 95% CI: 0.24–0.48, *p* < 0.001), and retrorectus placement (IRR: 0.303, 95% CI: 0.21–0.44, *p* < 0.001). Supplementary analysis showed that light-weight mesh was incorrectly identified as medium-weight mesh in 40% of cases (Table 6).

**Table 6:** Comparative analysis of diagnostic accuracy between radiologists and surgeons

Parameter	Current Study (Radiologists, *N* = 40)	Messer et al. (Surgeons, *N* = 22)	Blake et al. (Surgeons, *N* = 15)
Overall mesh plane identification accuracy	39.2%	-	36.5%*
Intraperitoneal/IPOM accuracy	53.8%	-	22%
Retrorectus/TAR accuracy	18.8% (with TAR), 16.9% (without TAR)	-	42% (Open TAR)
Mesh type identification accuracy	43.5%	44.5%	-
Heavy-weight mesh identification	52.5%	35.4%	-
Medium-weight mesh identification	30.6%	51.8%	-
Interrater reliability coefficient	0.127 (AC1)	0.428 (95% CI 0.356–0.503)	0.23 (95% CI 0.15–0.302)
Repeatability (R)	ICC - Mesh Type: 0.360 ICC - Mesh Plane: 0.270	0.053 (95% CI 0–0.119.119)	0.046 (CI 0–0.14.14)
Negative control identification accuracy	55.8%	87.9%	67%

Table 6. Direct comparison of our findings with those of Messer and Blake, highlighting the consistent challenges across both radiologist and surgeon specialties

*Average calculated from reported success rates for different approaches

Interrater reliability analysis (Table 5, Part B) demonstrated poor agreement, with Gwet’s AC1 of 0.127 (95% CI: 0.073–0.180, *p* = 0.0001), and substantial interobserver variability, with a variance of 0.480 (95% CI: 0.229–1.008, *p* = 0.008). Individual performance metrics indicated greater consistency in mesh type identification (IRR: 54.97) compared to mesh plane identification (IRR: 37.35), though both demonstrated poor repeatability, with ICCs of 0.36 and 0.27, respectively.

Multivariate analysis revealed that clinical experience did not significantly predict diagnostic accuracy, while radiologist confidence showed a strong positive correlation with performance. The majority of radiologists (72.5%) reported intermediate-low confidence (level 2/5), while 15% reported high confidence (levels 3–4/5), and 12.5% indicated low confidence (level 1/5). Higher confidence levels were significantly associated with improved accuracy, with the highest confidence level showing the strongest correlation (OR 4.55, 95% CI 1.94–10.95, *p* < 0.001).

Subgroup analysis by experience revealed a non-linear relationship with diagnostic accuracy. Radiologists with 5–7 years experience showed the highest median performance (8/18 correct) but greatest variability (range: 1–12), while those with 1 year performed similarly (median: 7/18, range: 2–12). The most experienced group (7–10 years) showed the lowest median accuracy (4/18, range: 3–6).

## Discussion

Our study suggests that radiologists face considerable challenges in interpreting post-ventral hernia repair CT scans. Despite specialized training and detailed guidance on mesh characteristics and anatomical planes, radiologists achieved only 39.2% accuracy in identifying mesh planes, with striking deficiencies in recognizing retrorectus (21.3%) and preperitoneal placements (22.5%), though they performed better with intraperitoneal placements (53.8%) and negative controls (55.8%). Mesh type identification proved similarly challenging at 43.5% overall accuracy, with heavy-weight mesh (52.5%) more readily identified than medium-weight and light-weight alternatives (30.6% each). Poor interrater reliability (Gwet’s AC1 = 0.127) and substantial between-radiologist variability (variance = 0.480) resulted in frequent misidentifications even when consensus was reached.

The ability to identify mesh type and placement has significant clinical implications. Understanding whether a previous repair used heavy-weight or medium-weight mesh can guide decision-making regarding potential mesh removal. In our experience, medium-weight mesh tends to integrate well with surrounding tissues, making removal more challenging, while heavy-weight mesh often maintains a more distinct plane. Knowledge of mesh type also offers insight into potential failure mechanisms. For instance, medium-weight mesh has higher fracture potential, and distinguishing between central mesh fracture versus fixation failure is essential for appropriate surgical planning [12]. Furthermore, accurate identification of mesh planes allows surgeons to select undissected planes for re-operative approaches, reducing surgical complexity and potential complications [4, 9, 10].

The diagnostic limitations we observed appear to be part of a broader pattern affecting the interpretation of post-VHR CT scans across medical specialties. Our findings align with recent systematic evidence. Abdelsamad et al. (2025) conducted a meta-analysis of 26 studies examining mesh visualization, reporting CT-based visualization rates of only 48% (95% CI: 0–100%), remarkably consistent with our 43.5% mesh type identification accuracy. Their finding that MRI offered superior mesh visualization (73%) suggests potential alternative imaging strategies, though CT remains the primary modality due to accessibility and cost considerations [13–17]. Furthermore, our findings closely parallel those reported by Blake et al., who found that abdominal wall reconstruction surgeons correctly identified surgical approaches in only 36.5% of cases when interpreting post-VHR CT scans. Their study of 15 expert surgeons evaluating 21 CT scans revealed particularly poor identification rates for robotic approaches (22% for both IPOM and eTEP), with slightly better recognition of open TAR and Rives-Stoppa procedures (42%). The remarkably similar accuracy rates between radiologists in our study and surgeons in Blake’s cohort suggest that these interpretative challenges transcend specialty training. Similarly, Messer et al. examined surgeons’ ability to identify mesh types on CT and found an overall accuracy of just 44.5%, with heavy-weight synthetic mesh correctly identified only 35.4% of the time—closely mirroring our finding of 52.5% accuracy for HWSM. Interestingly, both studies reported comparable interrater reliability coefficients (0.23 in Blake’s study vs. 0.27 in ours), indicating consistent patterns of diagnostic uncertainty across professional boundaries [9].This diagnostic congruence across specialties reveals a fundamental challenge inherent to post-VHR imaging interpretation rather than a profession-specific limitation. To our knowledge, this is the first study evaluating radiologists’ ability to interpret post-VHR CT scans and identify mesh characteristics. This interventional study recruited 40 body radiologists from 16 international centers and provided them with specialized training on identifying mesh planes and types. Our methodology paralleled that of Blake et al., employing multiple readers evaluating standardized cases, though we expanded the reviewer cohort from 15 surgeons to 40 radiologists, enhancing statistical power. Unlike Messer’s focus on mesh types alone, our study evaluated both mesh types and planes, providing a more comprehensive assessment of diagnostic challenges [4, 9]. The similarity in accuracy rates between our radiologist cohort and both surgical cohorts despite methodological differences in reader selection, training approaches, and case presentation reinforces the conclusion that this limitation transcends specialty boundaries.

While the overall diagnostic accuracy was limited, an interesting association emerged between radiologist confidence and interpretive performance. Although 72.5% of radiologists reported intermediate to low confidence (level 2/5), those with higher confidence (level 4/5) demonstrated significantly greater accuracy (55.6%) compared to those with the lowest confidence (24.4%), with a progressive improvement observed across confidence levels (OR 4.55 for level 4, *p* < 0.001). Notably, years of clinical experience showed no correlation with diagnostic accuracy. Subgroup analysis revealed a non-linear relationship, with mid-career radiologists performing best while the most experienced group demonstrated the lowest accuracy. This pattern reinforces that post-VHR CT interpretation requires specialized domain knowledge rather than general radiological experience. However, an inherent challenge exists as abdominal wall reconstruction has evolved rapidly in recent years, with new surgical techniques, mesh materials, and approaches continuously developing. The knowledge gap between specialties is understandable given that many of these techniques and materials are relatively new to surgical practice and this information has not yet fully reached parallel disciplines. As Leeuwenburgh et al. demonstrated, targeted training with direct feedback significantly improved radiologists’ diagnostic accuracy for specific abdominal pathologies, highlighting how specialized knowledge can overcome diagnostic challenges [13].

Given these interpretative limitations across specialties, multidisciplinary team (MDT) approaches may represent a promising solution for improving diagnostic accuracy. Prior studies have underscored the clinical value of structured collaboration in enhancing clinical decision-making. Parag and Hardcastle et al. found that despite good interobserver agreement between neurosurgeons and radiologists in traumatic head CT interpretation (concordance rate 91.64%, kappa 0.78), this level of agreement was only possible through established diagnostic criteria and collaborative interpretation [14]. Li et al. conducted a propensity score-matched study of 236 colorectal cancer patients with liver metastases and found that those managed by MDT demonstrated significantly better overall survival (HR 0.550, 95% CI: 0.309–0.977, *p* = 0.041) despite having worse biological characteristics at baseline [15]. This survival benefit was attributed to more consistent application of evidence-based treatment protocols and superior coordination of complex care pathways, suggesting similar advantages could be realized in complex hernia management.

A structured implementation of MDT for complex hernia cases would involve regularly scheduled conferences where complex hernia cases are reviewed jointly by AWR surgeons and body radiologists before surgery. During these sessions, radiologists would focus on anatomical landmarks while surgeons would provide context from clinical examinations and skills, creating a comprehensive assessment that neither could achieve independently. Implementation could also include standardized presurgical CT reporting templates specifically for post-VHR patients, developed collaboratively between specialties, that highlight key features needed for surgical planning while acknowledging inherent diagnostic limitations. The value of such structured approaches is increasingly recognized. Parker et al. (2024) recently described their ‘Complex Hernia Bundle’ pathway, emphasizing that MDT review is ‘increasingly recognized as the path forward in management to optimize patients and improve outcomes.’ Their experiential report from a high-volume center reinforces our recommendation for institutionalized collaboration between radiologists and surgeons [18]. Blake et al. demonstrated that radiologist-surgeon collaboration significantly improved diagnostic reliability in post-hernia repair imaging, with interobserver agreement improving dramatically from κ = 0.44 to κ = 0.91 after consensus discussions, providing strong evidence that such collaborative interpretation could overcome the limitations we observed in individual specialty performance [9]. Parikh et al. (2017) further emphasized the importance of standardized reporting of key features including mesh plane, type, and surrounding anatomical relationships to guide surgical decision-making [11].

The value of MDT approaches extends beyond diagnostic accuracy to patient outcomes. Meta-analyses across surgical specialties have shown that MDT care reduces postoperative mortality (OR 0.67; 95% CI, 0.51–0.88; *p* = 0.004) and shortens length of stay. Within surgical oncology, radiologists’ participation in MDTs has evolved from providing diagnostic support to strategically influencing treatment planning, impacting clinical decisions in up to 36.9% of cases. Li et al.‘s study specifically demonstrated that patients with more complex presentations (multiple liver metastases, bilobar distribution, node-positive primary tumors) particularly benefited from the MDT approach, supporting the notion that patients with complex hernias would similarly benefit from multidisciplinary care [15]. Additionally, Balasubramaniam et al. (2015) documented that radiologists’ participation in surgical MDTs has been associated with improved treatment planning accuracy and reduced reoperations by 19%, reinforcing the potential value of this approach in hernia management [16].

Synthesizing these findings within the broader clinical context highlights several actionable implications. First, both surgeons and radiologists should interpret CT scans of complex abdominal wall reconstruction with caution and appropriate reliance on clinical history, physical findings, and prior operative reports. Second, multidisciplinary collaboration between radiologists and abdominal wall surgeons should be actively pursued and institutionalized to enhance diagnostic accuracy. Third, comprehensive and standardized operative documentation is critical to guide future reoperative planning and improve long-term surgical outcomes.

## Study limitations

This study has several limitations. **First**, the number of CT scans included was limited to 18, which may constrain the generalizability of the findings. However, diagnostic power was augmented by including 40 board-certified radiologists, resulting in a total of 720 independent CT interpretations. This large number of individual assessments allowed for robust interobserver analysis and statistical evaluation of diagnostic patterns. **Second**, radiologists reviewed scans via video recordings rather than dedicated workstations, which represents a departure from standard clinical workflow. While we provided scrollable videos in three orthogonal planes (axial, coronal, and sagittal) to partially mitigate this limitation, the inability to perform real-time multiplanar reformats, adjust window/level settings, or measure Hounsfield units may have negatively impacted diagnostic accuracy. This constraint, while necessary for standardization across 16 international centres, likely underestimates radiologists’ true diagnostic capabilities in clinical practice where full PACS functionality is available. **Third**, individual radiologists assessed each case only once, which precluded assessment of intra-observer consistency and limited evaluation of personal diagnostic stability over time, though multiple cases per category provided some insight into interpretive patterns. **Fourth**, this study’s exclusive evaluation of polypropylene meshes (heavy, medium, and light-weight) represents a significant limitation. Clinical practice increasingly utilizes composite meshes with anti-adhesive barriers (ePTFE, oxidized regenerated cellulose), biosynthetic materials, and various fixation devices including radiopaque tacks. These materials create distinct imaging signatures—composite barriers appear as discrete layers, while metallic fixation creates characteristic artifacts. Our findings may not generalize to these materials. Future studies should evaluate radiologist accuracy with the full spectrum of clinically used meshes and fixation methods to provide comprehensive guidance for image interpretation in recurrent hernia planning. **Fifth**, while all radiologists received standardized training, we did not implement formal competency testing to verify adequate knowledge acquisition before study participation. This may have contributed to performance variability, as we cannot confirm that all participants achieved a uniform understanding of mesh characteristics and anatomical planes. **Finally**, the use of a structured multiple-choice response format with predefined mesh plane categories may have introduced cueing bias or constrained nuanced diagnostic input. To address this, we administered a pre-assessment survey to evaluate baseline knowledge and confidence, allowing us to better interpret the influence of structured responses on overall performance.

## Conclusion

This study underscores radiologists’ difficulty in accurately interpreting post-VHR CT scans despite targeted instruction and subspecialty in body CT. The diagnostic constraints observed are not unique to radiology but rather reflect a broader challenge shared with AWR surgeons. These findings emphasize the need for enhanced collaboration in preoperative planning for complex abdominal wall reconstruction. Rather than relying solely on isolated radiologic interpretation, our results support implementing structured multidisciplinary approaches where surgeons and radiologists jointly review complex cases, sharing their complementary expertise to optimize diagnostic accuracy and surgical planning. When combined with improved operative documentation and standardized CT reporting protocols, such approaches may offer a better opportunity to enhance diagnostic precision and patient outcomes.

## Supplementary Information

Below is the link to the electronic supplementary material.

Supplementary Material 1 (DOCX 30.8 KB)

## Declarations

### Artificial Intelligence

No artificial intelligence tools were used in study design, data collection, analysis, or manuscript preparation. All writing and analysis represent original work by the authors.

## Abbreviations

AWR: Abdominal Wall Reconstruction
CT: Computed Tomography
HWSM: Heavy-weight Synthetic Mesh
MWSM: Medium-weight Synthetic Mesh
LWSM: Light-weight Synthetic Mesh
VHR: Ventral Hernia Repair
TAR: Transversus Abdominis Release
IRR: Incident Rate Ratio
ICC: Intraclass Correlation Coefficient
MDT: Multidisciplinary Team
IQR: Interquartile Range
OR: Odds Ratio
