# Supplementary material for: Evaluation of radiologists’ accuracy and interrater reliability when interpreting CT scans after ventral hernia repair
Source: Hernia. 2025 Oct 31;30(1):3. doi: 10.1007/s10029-025-03504-7 (PMC12578746; doi:10.1007/s10029-025-03504-7)
Supplement: Supplementary file 1 [file 10029_2025_3504_MOESM1_ESM.docx]

Supplementary Table 1: Detailed Case Characteristics (n=18)

| Case # | Type | Surgical Approach | Mesh Type | Mesh Brand | Weight (g/m²) | Time from Surgery to CT | CT Slice Thickness | Reconstruction Kernel |
| --- | --- | --- | --- | --- | --- | --- | --- | --- |
| 1 | VHR | Intraperitoneal | Heavy-weight | PROLENE® | 95 | 24 months | 5mm | Soft tissue |
| 2 | VHR | Intraperitoneal | Heavy-weight | PROLENE® | 95 | 23 months | 5mm | Soft tissue |
| 3 | VHR | Intraperitoneal | Medium-weight | Bard™ Soft | 43 | 24 months | 5mm | Soft tissue |
| 4 | VHR | Intraperitoneal | Light-weight | Ultrapro™ | 26 | 25 months | 5mm | Soft tissue |
| 5 | VHR | Retrorectus | Heavy-weight | PROLENE® | 95 | 24 months | 5mm | Soft tissue |
| 6 | VHR | Retrorectus | Medium-weight | Bard™ Soft | 43 | 24 months | 5mm | Soft tissue |
| 7 | VHR | Retromuscular (TAR) | Medium-weight | Bard™ Soft | 43 | 23 months | 5mm | Soft tissue |
| 8 | VHR | Retromuscular (TAR) | Light-weight | Ultrapro™ | 26 | 24 months | 5mm | Soft tissue |
| 9 | VHR | Preperitoneal | Heavy-weight | PROLENE® | 95 | 24 months | 5mm | Soft tissue |
| 10 | VHR | Preperitoneal | Medium-weight | Bard™ Soft | 43 | 25 months | 5mm | Soft tissue |
| 11 | VHR | Preperitoneal | Light-weight | Ultrapro™ | 26 | 24 months | 5mm | Soft tissue |
| 12 | VHR | Preperitoneal | Light-weight | Ultrapro™ | 26 | 24 months | 5mm | Soft tissue |
| 13 | Control | Laparotomy only | N/A | N/A | N/A | 3 months | 5mm | Soft tissue |
| 14 | Control | Laparotomy only | N/A | N/A | N/A | 4 months | 5mm | Soft tissue |
| 15 | Control | Laparotomy only | N/A | N/A | N/A | 6 months | 5mm | Soft tissue |
| 16 | Control | No surgery | N/A | N/A | N/A | N/A | 5mm | Soft tissue |
| 17 | Control | No surgery | N/A | N/A | N/A | N/A | 5mm | Soft tissue |
| 18 | Control | No surgery | N/A | N/A | N/A | N/A | 5mm | Soft tissue |

Supplementary File 1. PowerPoint file containing instructions and explanations for the pre-test

|  |
| --- |

Download the attached PowerPoint presentation via Google Slides by clicking on the following link: PowerPoint Presentation [https://docs.google.com/presentation/d/1kuqRN5WLplPgMg17jftjJTPDQxdV2PYw/edit?usp=drive_link&ouid=103007429770211531794&rtpof=true&sd=true](https://docs.google.com/presentation/d/1kuqRN5WLplPgMg17jftjJTPDQxdV2PYw/edit?usp=drive_link&ouid=103007429770211531794&rtpof=true&sd=true) 

To answer your questions, click on the next link. For your convenience, we suggest opening this link on your smartphone: Questionnaire [https://docs.google.com/forms/d/e/1FAIpQLScCOZeGly4GEi2tjyQtrxZH2IThEv1GmDiZsmQvz5-SXK325g/viewform?usp=sf_link](https://docs.google.com/forms/d/e/1FAIpQLScCOZeGly4GEi2tjyQtrxZH2IThEv1GmDiZsmQvz5-SXK325g/viewform?usp=sf_link)
